# Triterpenoid Saponin Biosynthetic Pathway Profiling and Candidate Gene Mining of the *Ilex asprella* Root Using RNA-Seq

**DOI:** 10.3390/ijms15045970

**Published:** 2014-04-09

**Authors:** Xiasheng Zheng, Hui Xu, Xinye Ma, Ruoting Zhan, Weiwen Chen

**Affiliations:** Research Center of Chinese Herbal Resource Science and Engineering, Key Laboratory of Chinese Medicinal Resource from *Lingnan*, Guangzhou University of Chinese Medicine, Guangzhou 510006, China; E-Mails: zheng.x.s1987@163.com (X.Z.); usermxy@163.com (X.M.); ruotingzhan@vip.163.com (R.Z.)

**Keywords:** *Ilex asprella*, triterpenoid saponins, biosynthesis, α-amyrin, RNA-Seq

## Abstract

*Ilex asprella*, which contains abundant α-amyrin type triterpenoid saponins, is an anti-influenza herbal drug widely used in south China. In this work, we first analysed the transcriptome of the *I. asprella* root using RNA-Seq, which provided a dataset for functional gene mining. mRNA was isolated from the total RNA of the *I. asprella* root and reverse-transcribed into cDNA. Then, the cDNA library was sequenced using an Illumina HiSeq™ 2000, which generated 55,028,452 clean reads. *De novo* assembly of these reads generated 51,865 unigenes, in which 39,269 unigenes were annotated (75.71% yield). According to the structures of the triterpenoid saponins of *I. asprella*, a putative biosynthetic pathway downstream of 2,3-oxidosqualene was proposed and candidate unigenes in the transcriptome data that were potentially involved in the pathway were screened using homology-based BLAST and phylogenetic analysis. Further amplification and functional analysis of these putative unigenes will provide insight into the biosynthesis of *Ilex* triterpenoid saponins.

## 1. Introduction

Triterpenoid saponins are a class of widespread secondary metabolites in the plant kingdom. Chemical composition of triterpenoid saponins includes a triterpene moiety as the sapogenin and one or more attached sugar moieties such as glycosyl, glucuronyl or xylosyl. Triterpenoid saponins have drawn the attention of researchers because of their diverse bioactivities, including anti-inflammatory [1], anti-cancer [2], anti-microbial [3], insecticidal and anti-herbivore [4,5] activities. Owing to their significant pharmacological activities, plants rich in triterpenoid saponins are usually exploited as drug sources. However, the availability of triterpenoid saponins is hampered due to their low yield of crude drug extraction and difficulties in purification. Understanding the biosynthesis of triterpenoid saponins could help solve this problem.

**Figure 1.**
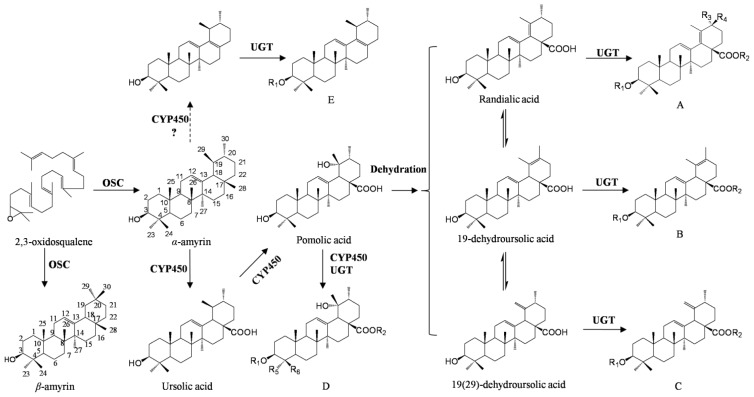
Putative triterpenoid saponins biosynthetic pathway downstream of 2,3-oxidosqualene in *I. asprella*.

*Ilex asprella*, a traditional herbal drug widely used in *Lingnan* area of China, is a major component of some popular cooling beverages and anti-influenza remedies, and with an annual consumption of over 10,000 tons, has great economic value. The most characteristic constituents of *I. asprella* are triterpenoid saponins, which show anti-cancer [6] and anti-virus activity [7]. To date, over 30 triterpenoid saponins have been isolated from the *I. asprella* leaves and roots (see Figure 1 and Table 1) [7–11]. These compounds can be classified into two main types: α-amyrin and β-amyrin. β-Amyrin, which is an oleanane, is a major configuration of pentacyclic triterpenoids, whereas α-amyrin, which is an ursane, is the isomer of β-amyrin but with a different location for C29 [12]. Interestingly, most of the triterpenoid saponins that were isolated from *I. asprella* roots were of the α-amyrin type (summarised in Table 1), except for one, which was of the β-amyrin type.

**Figure 2.**
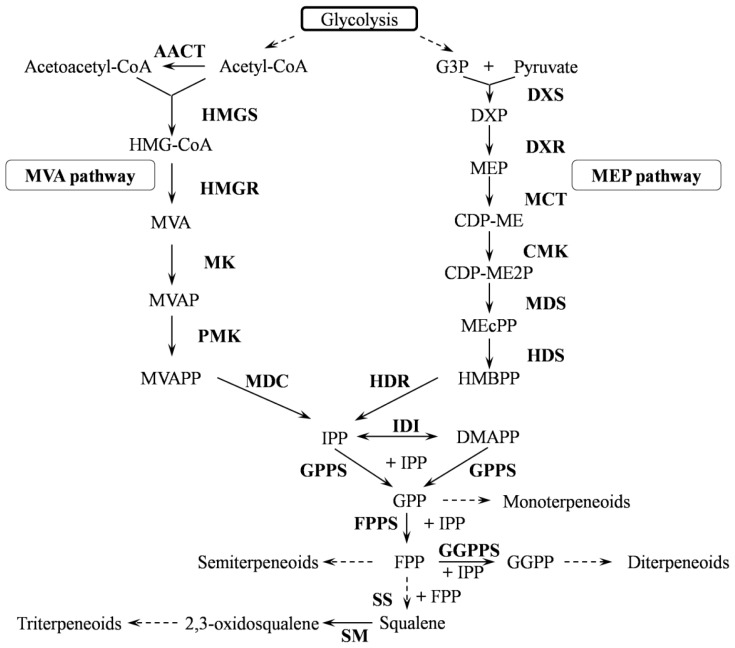
Terpenoid backbone biosynthetic pathway.

Over the past few years, the biosynthesis of triterpenoid saponins in some economically important plants, such as *Glycine max* [13] and *Panax ginseng* [14], has been studied. A biosynthetic pathway starting with the cyclisation of 2,3-oxidosqualene was suggested and involves three main steps: (i) cyclisation of 2,3-oxidosqualene catalysed by oxidosqualene cyclase (OSCs, EC 5.4.99.x); (ii) oxidative modification at various positions of the skeleton mediated by cytochromes P450 (P450s, EC 1.14.x.x); and (iii) glycosylation of the decorated skeleton catalysed by family 1 uridine diphosphate glycosyltransferases (UGTs, EC 2.4.1.x). Accordingly, a hypothetical biosynthetic pathway of triterpenoid saponins in *I. asprella* is described in Figure 1. The biosynthetic pathway upstream of 2,3-oxidosqualene is believed to be the mevalonic acid (MVA) pathway in the cytosol, although evidence exists for crosstalk between the MVA and the methylerythritol phosphate (MEP) pathways [15] (see Figure 2, which is adapted from the KEGG map00900 and modified according to the present study).

The identification of genes involved in the biosynthetic pathway of terpenoid saponins has been achieved by using many different techniques, including the next-generation sequencing technology (NGS). A recently developed technique called RNA Sequencing (RNA-Seq) for transcriptome profiling using NGS techique has shown great potential for functional gene mining for non-model plants [16,17] and can help in the discovery of rare transcripts in the transcriptome owing to its great sequencing depth. Since no appropriate reference is available for the non-model plants, *de novo* assembly is the only option for sequence assembly [16]. Therefore, RNA-seq utilising Illumina next-generation sequencing was used for the transcriptomic study of the *I. asprella* root and the detection of candidate genes involved in the triterpenoid saponin biosynthetic pathway as presented in this study.

## 2. Results and Discussion

### 2.1. RNA-Seq Output, Sequence Assembly and Gene Annotation

#### 2.1.1. Transcriptome Sequencing Output and Sequence Assembly

Next-generation sequencing was performed on RNA extracted from the *I. asprella* root and provided 55,028,452 high-quality (HQ) reads out from 58,670,910 raw reads (a yield of 93.79%). The Q20 and GC percentages were 98.08% and 46.34%, respectively. *De novo* assembly of these HQ reads produced 110,049 contigs of 36,036,333 nucleotides (nt) and the average length of these contigs was 327 nt, with an N50 of 540 nt. Further assembly of these contigs generated 51,865 unigenes; and the mean length and N50 of the unigenes were 685 and 1028 nt, respectively. Furthermore, the 51,865 unigenes could be grouped into 16,517 distinct clusters and 35,348 distinct singletons, using homologous transcription cluster analysis. The distribution of contigs and unigenes is shown in Figure S1.

#### 2.1.2. Gene Expression Overview

To investigate the expression levels of the sequencing data, the FPKM (Fragments per kilobase of exon model per million mapped fragments) values were applied to normalise and evaluate each unigene. Statistics of the distribution of the FPKM values, listed in Table 2, showed that the expression level of most unigenes was between 1 and 10.

#### 2.1.3. Functional Annotation

The 51,865 unigenes were successfully annotated through comparison with the sequences in the major public databases. In total, 39,269 unigenes were annotated to at least one database, which accounted for 75.71% (see Table 3). For Gene Ontology annotation, 29,375 unigenes were mapped to 57 functional groups (see Figure S2), among which, 18,932 were involved in the “metabolic process”. Of the 12,860 unigenes that were assigned to the COG database, 656 belonged to the cluster “secondary metabolites biosynthesis, transport and catabolism” (see Figure S3). The KEGG annotation profiled the biological pathways that are active in *I. asprella* and 20,752 unigenes were mapped to 128 KEGG pathways. Moreover, 272 unigenes were assigned to five terpenoid-like biosynthesis processes. One hundred and two unigenes (0.49%) mapped to “terpenoid backbone biosynthesis”, 13 (0.06%) mapped to “Monoterpenoid biosynthesis”, 77 (0.37%) mapped to “Diterpenoid biosynthesis”, 30 (0.14%) mapped to “Sesquiterpenoid and triterpenoid biosynthesis” and 50 (0.24%) mapped to “Ubiquinone and other terpenoid-quinone biosynthesis”. Based on the annotation results, the candidate genes related to terpenoid backbone and triterpenoid synthesis were identified and discussed in detail.

### 2.2. Candidate Genes Involved in the Biosynthesis of Triterpenoid Saponins

#### 2.2.1. Terpenoid Backbone Biosynthesis

Terpenoids are derived from C5 isoprene units through a “head-to-tail” connection. The conjunction of a different number of C5 isoprene units brings about various intermediates, such as IPP (C5 unit), DMAPP (C5 unit), GPP (C10 unit), and FPP (15 unit), which form the carbon skeletons of the different terpenoids. IPP, along with its isomer, DMAPP, are important intermediates in terpenoid backbone formation; both intermediates can be synthesized through the MVA pathway in the cytoplasm, or the MEP pathway in the plastid. Genes encoding all of the essential enzymes for both pathways were found in this transcriptome data, indicating that both pathways are active in the *I. asprella* roots.

**Figure 3.**
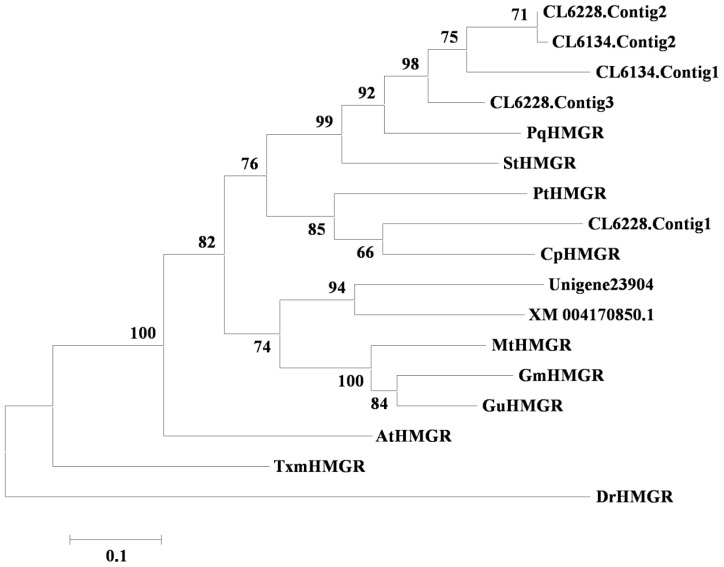
Phylogenetic tree of HMGRs. *Arabidopsis thaliana* AtHMGR (NM_127292), *Cucumis sativus* CsHMGR (XM_004170850), *Cyclocarya paliurus* CpHMGR (EU296534), *G. max* GmHMGR (XM_003547838), *Glycyrrhiza uralensis* GuHMGR (GQ845405), *Medicago truncatula* MtHMGR (XM_003629008), *P. quinquefolius* PqHMGR (FJ755158), *Populus trichocarpa* PtHMGR (XM_002313533), *Solanum tuberosum* StHMGR (NM_001288532), *Taxus x media* TxmHMGR (AY277740), Outgroup: *Danio rerio* DrHMGR (NM_001014292).

The MVA pathway is essential for the biosynthesis of sterols, sesquiterpenes and triterpenoids. Twenty-two unigenes in the *I. asprella* transcriptome, including four AACT genes, three HMGS genes, six HMGR genes, one MK gene, five PMK genes and three MDC genes (see Table S1), were identified to be involved in the MVA pathway. Among these enzymes, HMGR catalyses the conversion of HMG-CoA into MVA, which is an irreversible, two-step biochemical reaction that reduces the thioester group into a primary alcohol [18]. Therefore, HMGR is considered an important rate-limiting enzyme in the MVA pathway. In this study, the presence of six highly homologous genes implied that HMGR might be encoded by multiple genes in *I. asprella*. Together with other plant HMGRs, these six candidate unigenes were derived from a common ancestor (see Figure 3).

**Figure 4.**
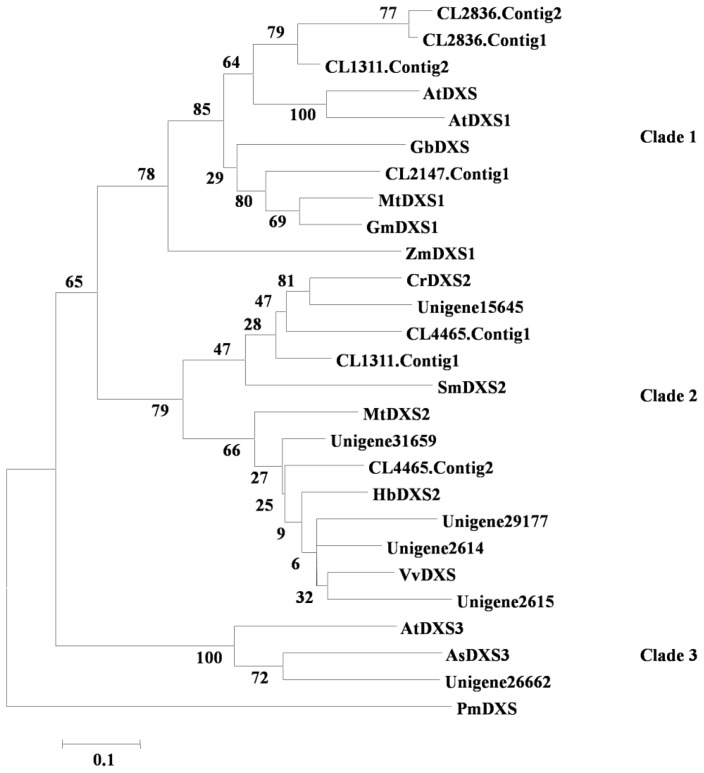
Phylogenetic tree of DXSs. This tree clearly shows that the distribution of the *I. asprella* sequences throughout the three clades of the tree. *Aquilaria sinensis* AsDXS3 (JX860325), *A. thaliana* AtDXS (NM_117647), AtDXS1 (NM_113045), AtDXS3 (NM_121176), *Catharanthus roseus* CrDXS2 (DQ848672), *Ginko biloba* GbDXS (AY505128), *G. max* GmDXS1 (NM_001249141), *Hevea brasiliensis* HbDXS2 (DQ473433), *M. truncatula* MtDXS1 (AJ430047), *Salvia miltiorrhiza* SmDXS2 (FJ643618), *Vitis vinifera* VvDXS (XM_002266889), *Zea mays* ZmDXS1 (NM_001164333), Outgroup: *Perkinsus marinus* PmDXS (AB284361).

Monoterpenes and diterpenes are synthesised through the MEP pathway, and 23 unigenes encoding enzymes involved in this pathway, including 13 DXS genes, four DXR genes, two MDS genes and one each of MCT, CMK, HDS and HDR (see Table S2), were found in the transcriptome. DXSs catalyse the formation of 1-deoxy-d-xylulose 5-phosphate through the condensation of pyruvate and glyceraldehydes-3-phosphate. The DXS genes can be classified into three clades: the DXS1 clade is involved in primary metabolism; the DXS2 clade is responsible for secondary terpenoid biosynthesis; and the recently elucidated DXS3 clade is involved in the biosynthesis of products that are essential for plant survival but expressed at a low level [19]. Further classification was predicted for these 13 genes with other defined plant DXSs using phylogenetic analysis (see Figure 4). The results showed that four unigenes, including CL2147.contig1, CL1311.contig2 and CL2836.cotig1 and 2, were grouped into the DXS1 clade; unigene26662 was the only unigene grouped into the DXS3 clade; and the remaining eight unigenes were grouped into the DXS2 clade.

Both MVA and MEP pathways produce the C5 unit IPP, which can be transformed into its isomer, DMAPP, by IDI (Isopentenyl diphosphate isomerase). Meanwhile, IPP and DMAPP are assembled into GPP, FPP and GGPP by a series of prenyl transferases, including GPPS, FPPS and GGPPS. FPP is an important intermediate of triterpenoid biosynthesis. Two units of FPP join in a “tail-to-tail” fashion, catalysed by squalene synthase (SS), to yield the hydrocarbon squalene. Subsequently, squalene is oxidised by squalene monooxygenase (SM) with the cofactors O_2_ and NADPH to give rise to another important precursor, 2,3-oxidosqualene. We found two IDI genes (Unigene3767, Unigene3833), three GPS genes (Unigene837 and CL7170.Contig1 and 2), four FPPS genes (CL2187.Contig1 to 4), seven GGPPS genes (CL4542.Contig1, CL6970.Contig1, Unigene10539, Unigene11301, Unigene17743, Unigene27823 and Unigene8778), three SS genes (CL3649.Contig1 to 3) and seven SM genes (CL3649.Contig1 to 3, Unigene14310, Unigene15274, Unigene18579 and Unigene1988) in the *I. asprella* transcriptome (see Table S3).

#### 2.2.2. Amyrin Synthaes

**Figure 5.**
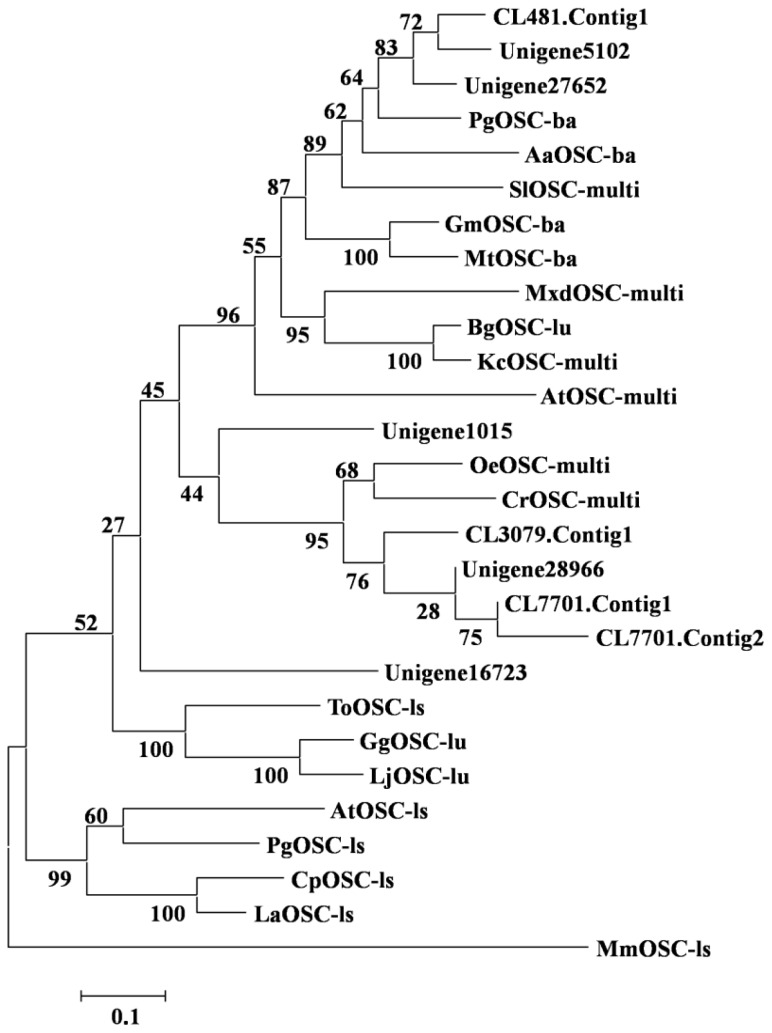
Phylogenetic tree of OSCs. The tree illustrates the likely gene function of 19 characterized OSC genes and nine candidate OSC unigenes found in this study. *A. thaliana* AtOSC-multi (NM_106497), *A. thaliana* AtOSC-ls (NM_114382), *Artemisia annua* AaOSC-ba (EU330197), *Bruguiera gymnorhiza* BgOSC-lu (AB289586), *C. roseus* CrOSC-multi (JN991165), *Cucurbita pepo* CpOSC-ls (AB116239), *G. max* GmOSC-ba (AY095999), *G. glabra* GgOSC-lu (AB116228), *Kandelia candel* KcOSC-multi (AB257507), *Lotus japonicas* LjOSC-lu (AB181245), *Luffa aegyptiaca* LaOSC-ls (AB033335), *Malus x domestica* MxdOSC-multi (FJ032006), *M. truncatula* MtOSC-ba (AJ430607), *Olea europaea* OeOSC-multi (AB291240), *P. ginseng* PgOSC-ba (AB009030), *P. ginseng* PgOSC-lu (AB009031), *Solanum lycopersicum* SlOSC-multi (HQ266580), *Taraxacum officinale* ToOSC-ls (AB025345), Outgroup: *Mus musculus* MmOSC (NM_146006); ba is for β-amyrin synthase, multi is for multifunctional OSC gene, ls is for lanosterol synthase, lu is for lupeol synthase.

**Figure 6.**
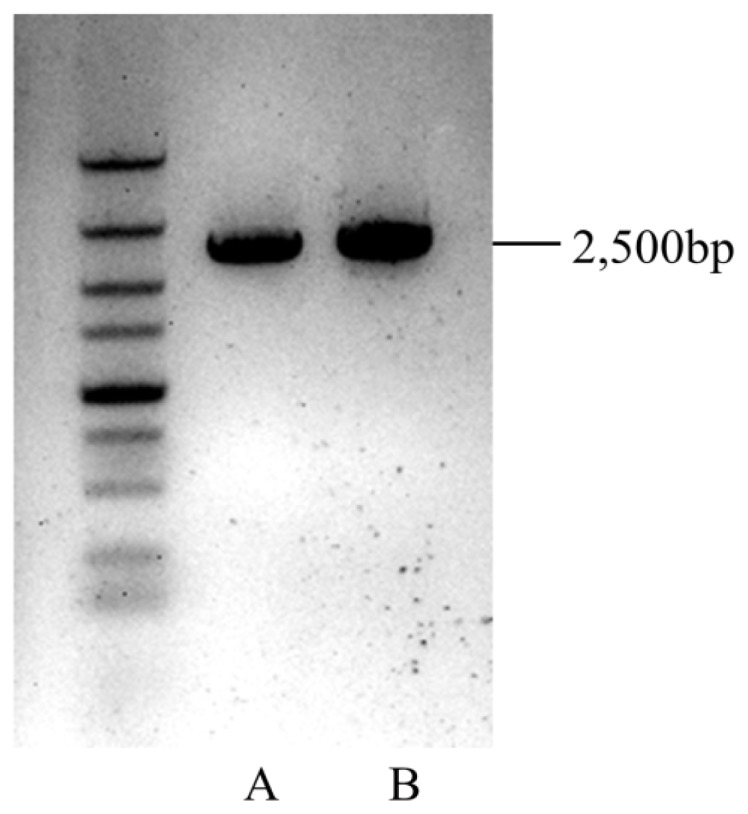
PCR products of amplified CL3079.contig1 (A) and CL481.contig1 (B).

As previously described, OSCs catalyse the cyclisation of 2,3-oxidosqualene to form a variety of triterpene skeletons [20], including phytosterol, dammarane, lupane and oleane (β-amyrin) [21]. This step is thus a critical branching point for phytosterol and triterpenoid biosynthesis. Over fifty different OSCs have been cloned from various plant species [22]. Among those OSCs, amyrin synthase catalyses the cyclisation of 2,3-oxidosqualene into α-amyrin and β-amyrin, resulting in a chair-chair-chair-boat conformation. Nine unigenes were identified to be amyrin synthase genes in this study (see Table S4). In addition, phylogenetic analysis of these nine unigenes indicated that they exhibit close homologous relationships with β-amyrin synthases and multifunctional amyrin synthases (see Figure 5). This prediction was supported by the presence of α-amyrin and β-amyrin type triterpenoids in the *I. asprella* roots. Among the nine candidates, CL3079.Contig1 and CL481.Contig1 were found to contain a full-length cDNA, including start and stop codons and a polyA signal, using the online tool GENSCAN. To confirm the gene sequences, primers were designed to anneal around the predicted start and stop codons of the two genes, resulting in an expected length of approximately 2500 base pairs (bp). Both genes were successfully amplified from cDNA generated from a different *I. asprella* root sample (see Figure 6, the sequencing result is shown in Figure S4) and will be characterised in the future.

The gene on CL3079.Contig1 (designated as *IaAS1*) has a length of 2271 bp encoding 756 amino acids, showing 82% amino acid sequence identity to the mixed amyrin synthase CrAS from *Catharanthus roseus*. In contrast, the gene on CL481.Contig1 (designated as *IaAS2*) is 2274 bp long encoding 757 amino acids, showing 86% amino acid sequence identity (Supplementary Figure S5) to the β-amyrin synthase AeAS from *Aralia elata* [23]. The QW repeat, DCTAE motif, as well as the MWCYCR motif, is present in both *IaAS1* and *IaAS2* [24–26]. Multiple alignment analysis of *IaAS1* and *IaAS2* with 19 ASs randomly selected from Genbank highlights some amino acid residues conserved in mixed AS. Among these, Glu46, the PVRXXE motif, Asn 157, Thr 263, Ile290, Leu402, Ile614 and Thr677 in *IaAS1* are likely candidates responsible for multiproduct nature exhibited by mixed AS, as these residues are located just near the QW repeat or conserved motif.

#### 2.2.3. P450s

Following the formation of amyrin, functional groups, such as hydroxyl and carboxyl, are introduced at different positions of the backbone, and this reaction is catalysed by the P450s (EC 1.14.x.x). This step contributes to increasing structural diversity [27,28]. As in the hypothesis described in Figure 1, CYP450s of C19-oxidase and C28-oxidase are essential for modifying α-amyrin during the formation of pomolic acid, which is an important intermediate compound. Dehydration of pomolic acid yields a second double bond, in addition to the first bond at C12–C13. This reaction might be catalysed spontaneously or by a dehydrase(s). Through either method, the dehydration of pomolic acid gives rise to randialic acid, 19-dehydrousolic acid and 19(29)-dehydrousolic acid, which are precursors of skeletons A, B and C, respectively. Moreover, isomerisation of these three precursors might take place spontaneously or mediated by isomerase. Further, C23-oxidase and C24-oxidase are necessary to form skeleton D. In brief, it is expected that these oxidisations are catalysed by P450s. The P450s is one of the largest and most diverse gene families in plants [29,30]. To date, only a few P450s have been identified as involved in triterpenoid biosynthesis. CYP93E1 from *G. max* was the first one characterised, and catalyses the C24-hydroxylation of β-amyrin [31]. CYP93E3 in liquorice exhibits a similar catalytic activity as CYP93E1 [32]; and CYP716A12 in *M. truncatula* and CYP716AL1 in *C. roseus* were characterised as multifunctional enzymes with β-amyrin 28-oxidase, α-amyrin 28-oxidase and lupeol 28-oxidase activities [27,33]. In the transcriptomic data, 269 unigenes were annotated to be P450s. BLASTp analysis using the above-mentioned characterised genes as queries against the transcriptome narrowed down the potential unigene number. Ten unigenes (CL1221.Contig1 to 3, CL3010.Contig1 to 4, unigene10591, unigene23155 and unigene25510) were highly homologous to CYP716A12 and CYP716AL1, with peptide sequence identities of more than 55%, which implies that they may belong to the same subfamily, according to the assignment of P450s to families and subfamilies developed by Nelson [34]. In addition, CL410.Contig1 exhibited 49.22% and 51.07% sequence identity to CYP93E1 and CYP93E3, respectively, which was coincident with the KEGG annotation. These P450s are shown in Table S5.

#### 2.2.4. UGTs

UGTs catalyse the transfer of glycosyl residues to the precursors that are decorated by P450s. The introduction of a glycosyl moiety to a triterpene increases its aqueous solubility, thus making it a triterpenoid saponin. UGTs catalyse the glucosylation of C3-hydroxyl and C28-carboxyl, which is essential to complete the triterpenoid saponin biosynthetic pathway in the *I. asprella* root. Like the P450s, UGTs constitute a large and diverse gene family. Sequences belonging to the same family and subfamily exhibit amino acid sequences identity >40% and >60%, respectively [35]. In the cDNA library of *I. asprella*, 335 unigenes were found to encode UGTs. Among them, five unigenes (CL679.Contig3, Unigene5668, Unigene29448, Unigene26225 and Unigene3060) exhibited high homology to UGT73C10 and UGT73C12 in *Barbarea vulgaris* (see Table S6), whose bioactivity is to catalyse the 3-*O*-glucosylation of oleanolic acid [36]; and one unigene (CL1465.Contig3) was homologous to UGT73F3 in *M. truncatula*, which catalyses the glucosylation of the C28-carboxy group of oleanane sapogenins [37]. Moreover, one unigene (Unigene82) was found to have the highest identity of 50.00% to the amino acid sequence of UGT74M1 in *Saponaria vaccaria* [38], which preferentially catalyses 28-*O*-glucosylation of oleanane-type sapogenins.

### 2.3. Discussion

Recently, interest in the biosynthesis of triterpenes has gradually increased because of their economical and scientific importance. A number of OSCs involved in the formation of triterpene carbon skeletons have been identified and characterized [39]. While monofunctional β-amyrin synthases and lupeol synthases were found, all the α-amyrin synthases identified so far are multifunctional and yield more than one product [33]. Many species of the *Ilex* genus of plants are rich in triterpenoid saponins, mostly of the α-amyrin type. The transcriptomic analysis of *I. asprella* has revealed a few AS candidate genes, and a close investigation into these candidates and their comparison with previously characterised AS genes would provide important knowledge of this gene family.

Unlike OSCs, the identification of new CYP450s and UGTs involved in the biosynthesis of triterpenoid saponins is beset with difficulties owing to the poor relationship between gene homology and functions of these two gene families. In this study, gene annotation provided a great number of putative CYP450s and UGTs. The candidate number was narrowed down to a few homologous unigenes by applying direct, homology-based screening of characterised genes. However, it is unknown whether these candidates are actually involved in the biosynthesis of triterpenoid saponins. Therefore, additional strategies should be engaged to identify credible candidate CYP450s and UGTs. The combination of exlicitor-induced expression regulation and co-expression analysis with OSC [33,37,40] would contribute to identification of the targeting CYP450s and UGTs in *I. aprella*.

Triterpenoid saponins were isolated from various tissues of *I. aprella* like roots and leaves, but this study is restricted to root tissue. A more thorough analysis of different plant tissues coupled with metabolomic data would help in building a global picture of *Ilex* triterpenoid biosynthesis and perhaps find novel candidate genes which would otherwise be difficult with sequence-homology-based searches.

## 3. Experimental Section

### 3.1. Plant Material and RNA Preparation

Two-year-old, potted *I. asprella* was collected from the Planting Base in Meizhou, Guangdong province, China. The *I. asprella* root was flushed under running tap water to remove soil and other attachments. After quick drying with bibulous papers, the root tissue was cut into approximately 1-mm-thick segments, snap frozen in liquid nitrogen and stored at −80 °C until further processing. Total RNA of the root was isolated using RNAiso Plus and RNAiso-mate for Plant Tissue (Takara, Dalian, China) following the product manual. The integrity, purity and concentration of the total RNA were analysed using agarose gel electrophoresis and ultraviolet spectroscopy.

### 3.2. cDNA Synthesis and Sequencing

Poly(A) mRNA was isolated from total RNA using Oligo (dT) beads, and then broken into short fragments using fragmentation buffer. Using these fragments as templates, random hexamer-primers were used to synthesise the first-stand cDNA. The second-strand cDNA was synthesised using GEX Second Strand buffer (10 μL), 25 mmol·L^−1^ dNTPs (1.2 μL), NRaseH (1 μL) and DNA polymerase I (5 μL). The short fragments of double-stranded cDNA were purified using the QiaQuick PCR extraction kit (Qiagen, Duesseldorf, NW, Germany) and eltuted with elution buffer for end repairing and adding of poly(A). Next, the short fragments were connected to sequencing adapters and purified by agarose gel electrophoresis. Suitable fragments were selected as templates for PCR amplification. Finally, the cDNA library was sequenced using an Illumina HiSeq™2000 (Illunima, San Diego, CA, USA).

### 3.3. Sequence Quality Control and Cleaning

The raw sequencing data of the cDNA library was transformed by base calling into raw reads, and stored in the FASTQ format. The sequence quality (sQ) was evaluated using the following formula: sQ = −10·lgE (E is the sequencing error rate). Raw reads (i) with a 3′ adaptor; (ii) with more than 5% uncertain nucleotides; and (iii) of low quality (Sq < 10 bases counted for more than 20% of the reads) were filtered out to generate clean reads. The clean reads were then used for further analysis and uploaded to the Sequence Read Archive (SRA) at NCBI with the accession number SRP035767.

### 3.4. Sequence Assembly

*De novo* assembly was performed using the short reads assembling program Trinity [41], which combines reads with a certain length of overlap to form longer fragments called contigs. Then, the reads were mapped back to these contigs. Furthermore, the contigs were assembled using Trinity to generate sequences that could not be extended on either end, which are defined as unigenes. Using homologous transcription cluster analysis, the unigenes were classified into two groups: unigenes with homologies higher than 70% were designed as clusters (initialled with CL, numbered with the gene family), while sole unigenes were designed as singletons (initialled with Unigene).

The assembled unique sequences were aligned to protein databases in the following order: Non-redundant (Nr) protein database, the Swiss-Prot protein database, the Kyoto Encyclopedia of Genes and Genomes (KEGG) database and the Cluster of Orthologous Groups of proteins (COG) database, by applying BLASTx (*E* value threshold set at 10^−5^). Sequence hits in a former database would not advance to a search against the next database. The CDS of the unigenes were extracted and translated into peptide sequences. Based on the BLAST results, the sequence direction was determined. For unigenes with uncertain direction after BLAST analysis, a statistical Hidden Markov Model (HMM) program, ESTscan [42], was introduced to help determine the sequence direction. Sequences whose directions could not be predicted using the ESTscan program were assigned their initial assembled sequence direction.

### 3.5. Gene Annotation

First, the unigene sequences were aligned by applying BLASTx against protein databases, including Nr, Swiss-Prot, KEGG and COG (*E* value threshold set at 10^−5^), and then using BLASTn against the Nucleotide (Nt) database (*E* value threshold set at 10^−5^). Proteins with the highest sequence similarity with the given unigenes along with their protein functional annotations were retrieved. Gene Ontology (GO) functional annotation of the unigenes was obtained along with the Nr annotation. The noted unigenes were assigned to GO categories for Molecular Function, Biology Process and Cellular Component using the Blast2GO program [43]. To portray the distribution of the functions of these noted unigenes, the WEGO program [44] was applied to classify the GO terms.

### 3.6. Gene Expression Analysis

The gene expression levels were analyzed by quantifying the read abundance observed. Paired-end reads mapped to a common contig were normalized by calculating FPKM values [45] for each contig by the formula followed: FPKM = (1,000,000·*C*)/(*N*·*L*·1000), where *C* is the number of fragments that uniquely aligned to an objective gene, *N* is the total number of fragments that uniquely aligned to all genes, and *L* is the number of bases in the objective gene.

### 3.7. Homology-Based Gene Discovery

Protein sequences of 4 characterized CYP450s and 4 UGTs, which were reported to be involved in the triterpenoid saponin biosynthetic pathway, were selected as objectives to run BLASTp analysis against the protein sequences that were translated from the raw reads data (*E* value threshold set at 10^−5^).

### 3.8. Phylogenetic Analysis

MEGA 6.05 was applied to perform the phylogenetic analysis of the nucleotide sequences of the target genes using the Maximum Likelihood method [46]. The reliability of all trees was evaluated using the bootstrap re-sampling method with 1000 replications.

### 3.9. Amplification of 2 OSC Genes

Total RNA was extracted from a different plant using the previously described method. Then, cDNA was synthesised using the PrimeScript™ RT-PCR Kit (TAKARA, Dalian, China) following the product manual. The primers for amplifying the complete CDS of CL3079.contig1 and CL481.contig1 were designed as followed:

for CL3079.contig1, Forward: TCTCTCTGTGTTTATGGGTA (5′→3′) and reverse: GAACACTGAAGGATACAAAC (5′→3′).
for CL481.contig1, Forward: GCCACAGTTATCTTCGTATT (5′→3′), and reverse: CATACTTCAAGGACCTCAAA (5′→3′).

The Polymerase Chain Reaction (PCR) contained 10 μL of PrimeSTAR Max DNA Polymerase (TAKARA, Dalian, China), 0.4 μL of each primer (10 mM), 1 μL of cDNA from the *I. asprella* root and water up to 20 μL. The amplification reaction was performed using the following temperature procedure: 98 °C for 2 min; 30 cycles of 98 °C for 10 s, 50 °C for 15 s, 72 °C for 15 s; and 72 °C for 5 min. Subsequently, 5 μL of the PCR product was mixed with 1 μL of 6× loading buffer, visualized using 1% agarose gel electrophoresis with Goldview dye (120 V for 12 min). Nucleotide sequencing was carried out by BGI Co., Lit (BGI, Beijing, China).

## 4. Conclusions

The transcriptome of the *I. asprella* root was obtained using RNA-Seq, resulting in many unigenes. The unigene dataset that was generated in this study provides a significant resource for further molecular studies of *I. asprella*, especially for characterising candidate genes in the biosynthetic pathways of triterpenoid saponins. Using appropriate approaches, a series of candidate genes were identified and were consequently analysed for expression patterns and phylogenetic relationships. A comprehensive bioinformatics analysis contributed to a better understanding of the candidate genes and to a reliable design for further research. The putative genes identified in *I. asprella* will be cloned and characterised in further studies.

## Supplementary Information



## Figures and Tables

**Table 1.** Main triterpenoid saponins isolated from the roots of *I. asprella*.

Triterpene skeleton	R_1_	R_2_	R_3_	R_4_	R_5_	R_6_	References
A *	H	H	CH_3_	H	-	-	[8]
	Xyl	Glc	CH_3_	H	-	-	[8]
	Xyl	Glc	H	CH_3_	-	-	[8]
	Glucuronic	Glc	H	CH_3_	-	-	[9]
	Glucuronic	Glc	CH_3_	H	-	-	[9]

B	H	H	-	-	-	-	[8]
	Xyl	Glc	-	-	-	-	[8]
	Glucuronic	Glc	-	-	-	-	[9]

C	Glucuronic acid methyl ester	Glucuronic	-	-	-	-	[9]

D	H	H	-	-	CH_3_	CH_3_	[10]
	H	Glc	-	-	CH_3_	CH_3_	[8]
	SO_3_Na	Glc	-	-	CH_3_	CH_3_	[8]
	Xyl	H	-	-	CH_3_	CH_3_	[8]
	Xyl	Glc	-	-	CH_3_	CH_3_	[8]
	Glucuronic	H	-	-	CH_3_	CH_3_	[9]
	Glucuronic-3-OSO_3_Na	Glc	-	-	CH_3_	CH_3_	[9]
	Glucuronic-3-OSO_3_Na	H	-	-	CH_3_	CH_3_	[9]
	Glucuronic	Glc	-	-	CH_3_	CH_3_	[9]
	Xyl-3-OSO_3_H	Glc	-	-	CH_3_	CH_3_	[7]
	Xyl-3-OSO_3_H	H	-	-	CH_3_	CH_3_	[7]
	Xyl(2-1)Glc(2-1)Rha	H	-	-	CH_3_	CH_3_	[7]
	Ara	Glc	-	-	CH_3_	CH_3_	[7]
	Xyl	Glc	-	-	CH_3_	CH_3_	[7]
	Ara(2-1)Glc	H	-	-	CH_3_	CH_3_	[7]
	H	Glc	-	-	CH_3_	COOH	[7]
	H	H	-	-	CH_3_	COOH	[7]
	Xyl	Glc	-	-	CH_2_OH	CH_3_	[7]

E	H	-	-	-	-	-	[10]
	Xyl	-	-	-	-	-	[10]

* The triterpene skeleton configurations are corresponded to Figure 1.

**Table 2.** FPKM values distribution.

Value of FPKM	Count	Proportion/%
>0	50,879	98.10
>1	46,426	89.51
>10	12,650	24.39
>100	1475	2.84
>1000	102	0.20

**Table 3.** Unigenes mapped to the public databases.

Public database	No. of matched unigenes	Annotation percentage/%
Nr	37,674	72.64
Nt	32,994	63.62
Swiss-Prot	22,661	43.69
KEGG	20,752	40.01
COG	12,860	24.80
GO	29,375	56.64
Total	39,269	75.71
