# Supplementary material for: Triterpenoid Saponin Biosynthetic Pathway Profiling and Candidate Gene Mining of the Ilex asprella Root Using RNA-Seq
Source: Int J Mol Sci. 2014 Apr 9;15(4):5970–87. doi: 10.3390/ijms15045970 (PMC4013608; doi:10.3390/ijms15045970)
Supplement: Supplementary file 1 [file ijms-15-05970-s001.pdf]

Supplementary Information

Figure S1. Distribution of contigs and unigenes.

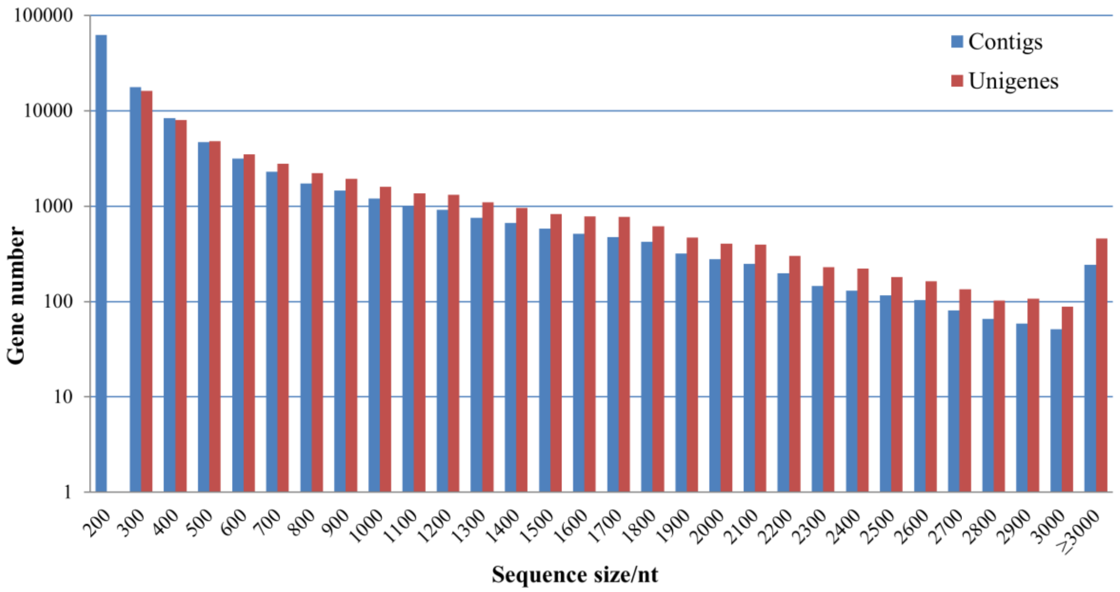

Figure S2. GO annotation.

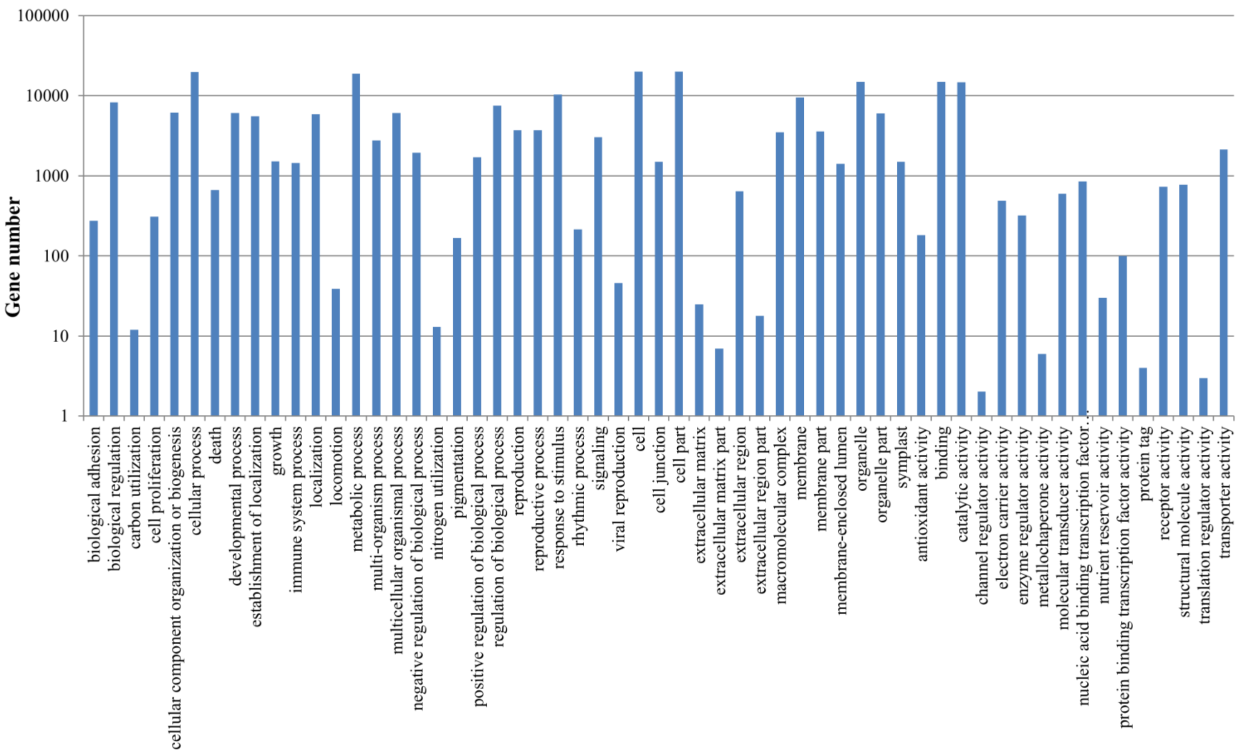

Figure S3. COG annotation.

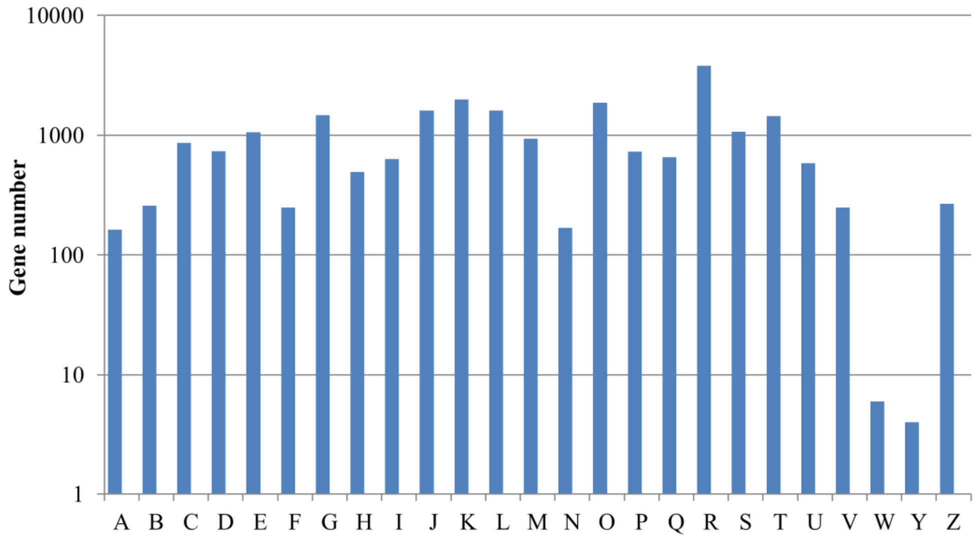

A:RNA processing and modification; B:Chromatin structure and dynamics; C:Energy production and conversion; D:Cell cycle control, cell division, chromosome partitioning; E:Amino acid transport and metabolism; F:Nucleotide transport and metabolism; G:Carbohydrate transport and metabolism; H:Coenzyme transport and metabolism; I:Lipid transport and metabolism; J:Translation, ribosomal structure and biogenesis; K:Transcription; L:Replication, recombination and repair; M:Cell wall/membrane/envelope biogenesis; N:Cell motility; O:Posttranslational modification, protein turnover, chaperones; P:Inorganic ion transport and metabolism; Q:Secondary metabolites biosynthesis, transport and catabolism; R:General function prediction only; S:Function unknown; T:Signal transduction mechanisms; U:Intracellular trafficking, secretion, and vesicular transport; V:Defense mechanisms; W:Extracellular structures; Y:Nuclear structure; Z:Cytoskeleton

Figure S4. Amino acid sequences of amplified CL3079.contig1 and CL481.contig1.

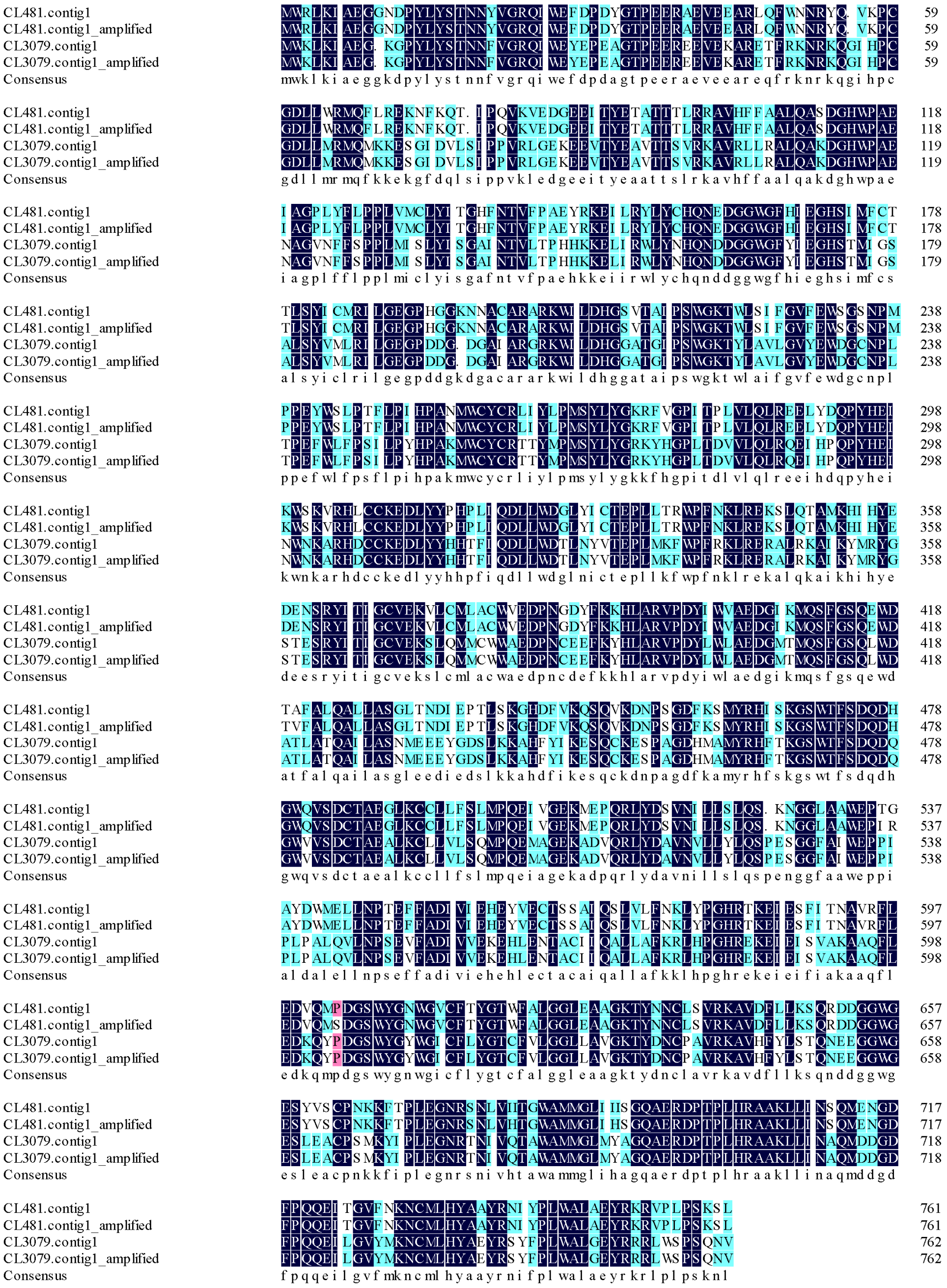

Figure S5. Multiple alignment analysis of *IaAS1* and *IaAS2* with 19 characterised ASs. Amyrin synthase from *Catharanthus roseus* [AEX99665.1], mixed amyrin synthase from *Eriobotrya japonica* [AFP95334.2], mixed amyrin synthase from *Catharanthus roseus* [AFJ19235.1], mixed amyrin synthase from *Olea europaea* [BAF63702.1], alpha/beta amyrin synthase from *Malus domestica* [ACM89977.1], mixed amyrin synthase from *Pisum sativum* [BAA97559.1], beta amyrin synthase from *Bruguiera gymnorhiza* [BAF80443.1], beta amyrin synthase from *Glycyrrhiza glabra* [Q9MB42.1], beta amyrin synthase from *Pisum sativum* [Q9LRH8.1], beta amyrin synthase 1 from *Panax ginseng* [O82140.1], beta amyrin synthase from *Solanum lycopersicum* [NP_001234604.1], beta amyrin synthase from *Glycyrrhiza uralensis* [ACV21067.1], beta amyrin synthase from

Artemisia annua [ACA13386.1], beta amyrin synthase from *Glycine max* [NP_001236591.1], beta amyrin synthase from *Gentiana straminea* [ACO24697.1], beta amyrin synthase from *Panax quinquefolius* [AGG09939.1], beta amyrin synthase from *Barbarea vulgaris* [AFF27506.1], beta amyrin synthase from *Arabidopsis thaliana* [BAG82628.1], beta amyrin synthase from *Withania somnifera* [AGA17940.1]. QW repeats (clear boxes), SDTAE motif (grey box), MFCYCR motif (stars), amino acid residues Glu46, Asn 157, Thr 263, Ile290, Leu402, Ile614 and Thr677 (arrow), and PVRXXE motif (bars).

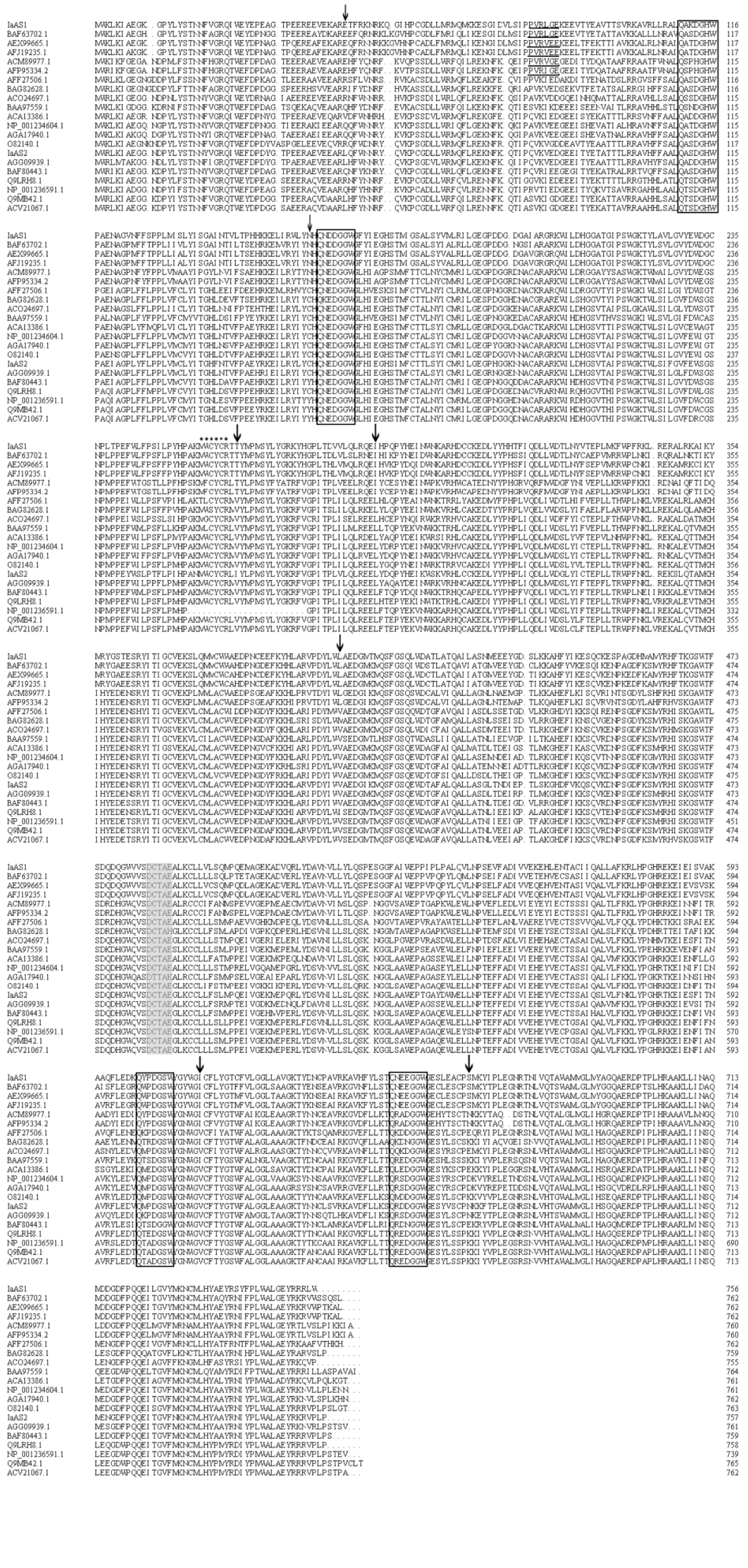

Table S1. Candidate genes of the MVA pathway in *Ilex asprella*.

Enzymes	E.C. No.	Gene ID	Length/nt	FPKM	Subject_id	Identity/%	*E* value
AACT	[2.3.1.9]	CL3187.Contig1	1722	145.1172	vvi:100240809	89.43	0
		CL3187.Contig2	724	11.0954	pop:POPTR_652350	83.47	7.00×10^{-113}
		Unigene10803	841	33.8032	pop:POPTR_652350	86.09	9.00×10^{-52}
		Unigene3311	235	5.5267	pop:POPTR_732472	92.31	6.00×10^{-34}
HMGS	[2.3.3.10]	CL6736.Contig1	1920	94.5512	vvi:100244379	87.98	0
		CL6736.Contig2	1502	7.6541	vvi:100244379	88.10	0
		Unigene700	348	5.5290	gmx:100780554	89.39	1.00×10^{-29}
HMGR	[1.1.1.34]	CL6134.Contig1	823	140.0404	vvi:100265082	62.50	5.00×10^{-79}
		CL6134.Contig2	1257	198.4557	vvi:100265082	70.94	4.00×10^{-149}
		CL6228.Contig1	2454	849.7300	vvi:100245191	78.87	0
		CL6228.Contig2	864	232.7174	gmx:100784330	92.38	1.00×10^{-114}
		CL6228.Contig3	761	155.4952	vvi:100245191	95.50	8.00×10^{-118}
MK	[2.7.1.36]	Unigene23904	2226	18.9298	vvi:100265082	79.93	0
		Unigene25854	1466	108.7389	rcu:RCOM_0573350	74.58	7.00×10^{-170}
PMK	[2.7.4.2]	CL6605.Contig1	204	3.7727	gmx:100810373	68.66	3.00×10^{-21}
		CL6605.Contig2	1653	16.8489	rcu:RCOM_0699810	80.24	0
		CL6605.Contig3	270	6.9481	pop:POPTR_712033	76.40	3.00×10^{-37}
		CL6605.Contig4	332	5.2159	vvi:100242124	87.96	3.00×10^{-51}
		Unigene29386	306	3.1439	pop:POPTR_739979	72.73	3.00×10^{-10}
MDC	[4.1.1.33]	CL6624.Contig1	1827	27.1185	vvi:100251686	84.17	0
		CL6624.Contig2	1747	44.6055	vvi:100251686	85.17	0
		Unigene32149	229	2.1005	gmx:100800990	58.82	5.00×10^{-10}

Table S2. Candidate genes of the MEP pathway in *Ilex asprella*.

Enzymes	E.C. No.	Gene ID	Length/nt	FPKM	Subject_id	Identity/%	*E* value
DXS	[2.2.1.7]	CL1311.Contig1	268	1.2564	vvi:100247834	95.45	1.00×10^{-46}
		CL1311.Contig2	1009	9.0579	vvi:100249323	93.15	0
		CL2147.Contig1	527	20.6283	mtr:MTR_4g118640	90.43	1.00×10^{-55}
		CL2836.Contig1	932	5.2128	vvi:100249323	82.35	7.00×10^{-125}
		CL2836.Contig2	588	0.0818	pop:POPTR_766757	94.62	2.00×10^{-97}
		CL4465.Contig1	296	3.0877	vvi:100247834	82.65	8.00×10^{-44}
		CL4465.Contig2	285	2.0254	gmx:100799983	91.58	3.00×10^{-46}
		Unigene15645	254	7.0070	vvi:100263394	80.95	7.00×10^{-25}
		Unigene2614	249	1.5455	vvi:100268079	90.24	1.00×10^{-38}
		Unigene2615	267	4.8643	vvi:100268079	80.68	9.00×10^{-38}
		Unigene26662	2794	28.1659	vvi:100252520	76.23	0
		Unigene29177	498	2.5114	vvi:100268079	77.30	4.00×10^{-67}
		Unigene31659	232	1.8660	mtr:MTR_8g068300	92.21	2.00×10^{-34}
DXR	[1.1.1.267]	CL5974.Contig1	1320	1.4212	vvi:100248516	89.55	0
		CL5974.Contig2	1252	0.4226	vvi:100248516	85.53	0
		Unigene20787	223	6.2555	vvi:100244507	75.76	7.00×10^{-9}
		Unigene22238	429	22.5375	osa:4326153	87.14	1.00×10^{-30}
MCT	[2.7.7.60]	Unigene12252	902	4.4796	pop:POPTR_1113320	87.20	9.00×10^{-56}
CMK	[2.7.1.148]	Unigene16595	1484	31.5064	vvi:100261596	79.03	4.00×10^{-180}
MDS	[4.6.1.12]	CL6364.Contig1	1216	15.9418	vvi:100250076	73.73	8.00×10^{-90}
		CL6364.Contig2	1085	2.0837	ath:AT1G63970	87.01	6.00×10^{-53}
HDS	[1.17.7.1]	Unigene24920	2610	120.9194	vvi:100257071	87.84	0
HDR	[1.17.1.2]	CL5975.Contig1	1714	24.6967	vvi:100267479	85.81	0

Table S3. Candidate genes of IDI, GPS, FPPS, GGPPS, SS and SM in *Ilex asprella*.

Enzymes	E.C. No.	Gene ID	Length/nt	FPKM	Subject_id	Identity/%	*E* value
IDI	[5.3.3.2]	Unigene3767	1266	128.1590	gmx:100783078	77.52	4.00×10^{-125}
		Unigene3833	2455	54.4703	gmx:100789313	27.83	3.00×10^{-7}
GPS	[2.5.1.10]	CL7170.Contig1	1691	5.6039	rcu:RCOM_0747390	67.06	2.00×10^{-180}
		CL7170.Contig2	1538	6.5054	rcu:RCOM_0747390	68.51	1.00×10^{-174}
		Unigene837	388	11.4057	rcu:RCOM_0747390	83.33	8.00×10^{-16}
FPPS	[2.5.1.1]	CL2187.Contig1	1549	58.4743	vvi:100232975	85.09	1.00×10^{-170}
		CL2187.Contig2	1469	2.2921	pop:POPTR_560081	87.29	3.00×10^{-153}
		CL2187.Contig3	1466	9.6467	vvi:100232975	87.68	6.00×10^{-175}
		CL2187.Contig4	746	1.2251	gmx:100802347	88.40	1.00×10^{-92}
GGPS	[2.5.1.10]	CL4542.Contig1	679	3.7547	pop:POPTR_649179	74.19	1.00×10^{-68}
		CL6970.Contig1	675	38.9095	rcu:RCOM_1373490	76.37	3.00×10^{-74}
		Unigene10539	1471	70.8946	vvi:100266842	71.20	2.00×10^{-146}
		Unigene11301	428	3.1469	rcu:RCOM_0082580	68.99	6.00×10^{-46}
		Unigene17743	696	9.1920	pop:POPTR_649179	74.19	1.00×10^{-68}
		Unigene27823	685	5.2667	vvi:100257359	49.28	1.00×10^{-27}
		Unigene8778	494	4.8687	vvi:100257359	84.15	6.00×10^{-74}
SS	[2.5.1.21]	CL6896.Contig1	1763	120.4605	vvi:100265798	87.41	0
		Unigene16826	234	12.9506	gmx:547940	82.76	2.00×10^{-7}
		Unigene25632	659	29.2702	vvi:100265798	73.33	1.00×10^{-25}
SM	[1.14.13.132]	CL3649.Contig1	322	1.7926	rcu:RCOM_0859900	87.85	1.00×10^{-52}
		CL3649.Contig2	1954	410.0517	vvi:100254037	83.75	0
		CL3649.Contig3	1286	6.1344	vvi:100254037	89.11	0
		Unigene14310	280	2.5769	vvi:100254037	94.92	2.00×10^{-27}
		Unigene15274	1032	15.1019	vvi:100254037	70.24	1.00×10^{-133}
		Unigene18579	803	34.8637	vvi:100265235	88.14	1.00×10^{-96}
		Unigene1988	331	5.6676	vvi:100265235	66.96	9.00×10^{-35}

Table S4. Candidate genes of OSC in *Ilex asprella*.

Gene ID	Length/nt	FPKM	Subject_id	Identity/%	*E* value
CL3079.Contig1	2707	163.4450	vvi:100259856	62.24	0
CL481.Contig1	2892	1.7797	vvi:100259856	86.00	0
CL7701.Contig1	234	2.2612	vvi:100259856	78.43	5.00×10^{-19}
CL7701.Contig2	217	0.4433	vvi:100259856	75.76	5.00×10^{-10}
Unigene1015	507	3.1309	vvi:100259856	64.02	2.00×10^{-62}
Unigene16723	654	5.0750	vvi:100259856	65.84	1.00×10^{-62}
Unigene27652	318	7.8658	vvi:100259856	82.69	9.00×10^{-51}
Unigene28966	289	4.3275	vvi:100259856	68.75	3.00×10^{-40}
Unigene5102	241	4.5907	ath:AT1G78955	81.25	3.00×10^{-39}

Table S5. Candidate genes of CYP450s involved in the triterpenoid saponins biosynthesis pathway of *Ilex asprella*.

Gene ID	Length/nt	FPKM	CYP716A12		CYP716AL1		CYP93E1		CYP93E3	
			Identity/%	*E* value	Identity/%	*E* value	Identity/%	*E* value	Identity/%	*E* value
CL1221.Contig1	269	2.1458	55.17	2.00×10^{-24}	60.23	3.00×10^{-27}				
CL1221.Contig2	1808	0.3725	55.96	1.00×10^{-152}	55.02	1.00×10^{-148}				
CL1221.Contig3	1799	0.7487	55.96	1.00×10^{-152}	55.02	1.00×10^{-148}				
CL3010.Contig1	1822	216.7510	76.54	0	87.02	0				
CL3010.Contig2	1619	221.7344	76.91	0	86.33	0				
CL3010.Contig3	1128	65.0746	70.59	7.00×10^{-144}	81.06	4.00×10^{-158}				
CL3010.Contig4	956	63.7004	68.82	5.00×10^{-107}	76.95	1.00×10^{-115}				
Unigene10591	360	106.2260	73.81	9.00×10^{-13}	80.00	2.00×10^{-13}				
Unigene23155	1197	13.8239	56.00	2.00×10^{-129}	57.22	4.00×10^{-132}				
Unigene25610	604	150.9968	80.74	5.00×10^{-65}	80.45	3.00×10^{-65}				
CL410.Contig1	1889	57.8807					49.22	2×10^{-147}	51.07	9.00×10^{-144}

Table S6. Candidate genes of UGTs involved in the triterpenoid saponins biosynthesis pathway of *Ilex asprella*.

Gene ID	Length/nt	FPKM	UGT73C10		UGT73C12		UGT73F3	
			Identity/%	*E* value	Identity/%	*E* value	Identity/%	*E* value
CL679.Contig3	254	1.8938	61.11	1.00×10^{-16}	60.53	6.00×10^{-22}	-	-
Unigene5668	240	2.6055	-	-	63.75	6.00×10^{-23}	-	-
Unigene29448	261	3.3174	-	-	62.16	4.00×10^{-7}	-	-
Unigene26225	766	24.4908	-	-	60.09	8.00×10^{-68}	-	-
Unigene3060	207	5.3447	-	-	60.00	1.00×10^{-16}	-	-
CL1465.Contig3	706	0.4769	-	-	-	-	60.26	2.00×10^{-43}

© 2014 by the authors; licensee MDPI, Basel, Switzerland. This article is an open access article distributed under the terms and conditions of the Creative Commons Attribution license (http://creativecommons.org/licenses/by/3.0/).
